# Estimating Economic Losses in Commercial Chicken Farms During COVID-19 Pandemic in Bangladesh: Lessons Learned for Future Pandemic

**DOI:** 10.1155/tbed/4935897

**Published:** 2025-06-22

**Authors:** Md Zulqarnine Ibne Noman, A. K. M. Dawlat Khan, Md Mehedi Hasan, Emama Amin, Md Arif Khan, Nabila Nujhat Chowdhury, Mohammed Mahmudul Hassan, Suman Das Gupta, Tahmina Shirin, Shusmita Dutta Choudhury, Ariful Islam

**Affiliations:** ^1^Zoonotic Disease Research Program, Institute of Epidemiology, Disease Control and Research (IEDCR), Mohakhali, Dhaka 1212, Bangladesh; ^2^Queensland Alliance for One Health Sciences, School of Veterinary Science, The University of Queensland, Brisbane 4343, Queensland, Australia; ^3^School of Agricultural, Environmental and Veterinary Sciences, Charles Sturt University, Wagga Wagga 2678, New South Wales, Australia; ^4^Gulbali Research Institute, Charles Sturt University, Wagga Wagga 2678, New South Wales, Australia

**Keywords:** consumer demand, economic cost, food security, poultry farms, supply chain

## Abstract

The COVID-19 pandemic has had a substantial impact on various economic sectors, including poultry production and trading in Bangladesh. We aimed to estimate the total economic losses and determine the causes behind these losses in commercial chicken farms during the COVID-19 in Bangladesh. We conducted a cross-sectional study using both qualitative and quantitative approaches across six districts from September to December 2021. The data collection involved semi-structured questionnaire interviews with 220 commercial poultry farmers and conducting in-depth interviews (IDIs) with 20 farm owners. We employed stepwise regression analysis to determine the optimal model for forecasting the average losses per farm caused by COVID-19. This model was built using the average reduced egg price, reduced bird price, and increased feed price of each individual farm. These averages were then used to predict the average farm loss, which was subsequently extrapolated to determine the total national loss. We estimated that the national loss in the small and medium-scale poultry sector during the COVID-19 lockdown amounted to 98.5 million USD, with the model predicting an average economic loss of $1407.6 per commercial farm. The majority (90%) of farmers experienced losses during this period. Majority of broiler (83.54%) and layer (80.65%) farmers, and approximately half of the Sonali farmers (54.10%) had to change their trading patterns or supply chains. After the pandemic period, about 33.33% of broilers, 31.03% of layer, and 45.90% of Sonali farmers managed to recover their losses. The poultry farmers encountered numerous challenges that impeded their ability to sell birds. These challenges resulted from transportation restrictions, widespread rumors leading to a sharp decline in demand, and significant losses from reduced egg and live bird prices. Consequently, some farmers were forced to close their farms, while others adapted by changing their trading patterns. To cope with the financial strain, some farmers resorted to obtaining loans from financial organizations, or seeking help from relatives, and a fortunate few received incentives from the government. Farmers suggested price monitoring, trainings, low-interest loans, and government incentives. Additionally, the formation of farmer's associations, exempting poultry from restrictions and lockdown, raising mass awareness, and including farmers' representatives in pandemic preparedness teams are deemed essential measures to safeguard the economic interests in any future pandemic crises. The marginal and small-scale poultry sectors in Bangladesh were severely impacted by the COVID-19 pandemic and the subsequent lockdown, with many farmers still struggling in recuperating their financial losses. It is imperative for the government to aids these farmers to support their contributions to protein supply and poverty alleviation in the community.

## 1. Introduction

The commercial poultry sector plays a pivotal role in the growing economy of Bangladesh. This rapidly growing sector reduces malnutrition, promotes agricultural growth, and meets the increasing domestic demand for eggs and meat. It employs approximately 8.5 million people, making it the second most important source of employment in Bangladesh [1, 2]. Poultry contributes 37% of the total meat production in Bangladesh and supplies 22%–27% of the total animal protein [3]. Additionally, the poultry sector accounts for 1.4% of the country's GDP overall [4]. Despite these positive contributions, the poultry industry in Bangladesh encountered significant challenges during the recent COVID-19 pandemic [5].

Bangladesh experienced community transmission of the coronavirus, with the first case detected on 8th March 2020 [6]. In response, the government declared a countrywide lockdown and general leave from 26th March to 30th June 2020, lasting for 97 days [7]. During this lockdown period, office holidays were enforced, public transport was suspended, gatherings and prayers were restricted, and all nonessential businesses and services were closed, with people instructed to stay at home [8]. Even after the initial lockdown, certain restrictions continued, such as limited seating on public transport, restricted movement at night, and limitations on business operating hours [9–12]. Throughout 2021, there were at least two additional lockdowns: from 28th June to 14th July (17 days) and from 23rd July to 5th August (14 days) [12]. Moreover, local governments-imposed lockdowns in various districts at different times. Consequently, Bangladesh experienced a range of restrictions imposed over a span of 1.5 years [13].

The enactment of COVID-19 response strategies has resulted in significant collateral health damage, an unprecedented economic crisis, and adverse impacts on multiple sectors, including the livestock production industry, particularly the commercial poultry sector in Bangladesh [5]. Similar to the situation in Bangladesh, the closure of schools, shops, markets, offices, and restaurants, along with restrictions on public gatherings and travel, has resulted in reduced demand for poultry chickens globally [14–16]. Management and treatment of poultry and livestock when sick was hampered during period of lockdown due to lack of availability of veterinarian [17]. Additionally, infections among farm owners and workers, as well as the fear of contracting COVID-19, have also led to farm closures [18]. Many of the farmers had to change their livelihood choices in order to cope with financial collapse of their poultry farms [19].

Furthermore, hatcheries increasing the price of day-old chicks (DOCs) during certain festivals and events has placed farmers in challenging situations. The rising cost of feed is another significant concern, and fluctuations in the demand for chicken and eggs during the pandemic have further impacted the financial well-being of farmers [3]. Poultry farmers experienced significant economic losses during the period of COVID-19 pandemic restrictions [20]. Despite these challenges, they employed their own coping strategies to survive and sustain their operations and developing their own perceptions for handling future pandemics [21, 22]. To gain insights into the true state of the poultry industry during the COVID-19 pandemic in Bangladesh, the objectives of our study were: (a) to estimate the economic losses of small and medium-scale commercial poultry farming during the pandemic, (b) to identify the reasons for these losses, and (c) to explore farmers' perspectives on economic loss, and gather their valuable recommendations based on their real-life experiences to better prepare for future pandemics.

## 2. Materials and Methods

### 2.1. Study Design and Location

This study utilized a descriptive cross-sectional design, incorporating both quantitative and qualitative approaches. Conducted from September to December 2021, the research spanned six districts in Bangladesh, including the capital city, Dhaka, and its surrounding districts: Gazipur, Narsingdi, Narayanganj, Manikganj, and Munshiganj. Six districts surrounding Dhaka city were selected due to the high concentration of poultry enterprises, including a large number of small- and medium-scale poultry farms, compared to other regions of Bangladesh. Additionally, these districts benefit from better transportation and communication networks with the capital, which facilitated the data collection process. Approximately 53.3% of poultry farms in Bangladesh are located in the Dhaka division, with around 24% situated specifically in Gazipur district [23]. A higher proportion of broiler and layer farms are concentrated in the Dhaka Division, and Sonali farms are predominantly found in the Dhaka region, as well as in the western and northern districts of the country [24]. As the capital of Bangladesh, Dhaka has a large population and thus accounts for a major share of the poultry economy. The six districts selected for our study, primarily supply poultry meat to meet Dhaka's demand [25]. Furthermore, the prices of poultry products, such as birds and eggs, as well as farming costs including feed, medicine, and DOCs, do not differ significantly across regions. Distribution and manufacturing of medicines, feed, DOCs, and other farming inputs are largely handled by private corporations, which tend to maintain uniform pricing across the country [20, 26], thereby ensuring the findings are representative and reflective of the broader conditions faced by poultry farmers in Bangladesh.

### 2.2. Sample Size and Data Collection

We purposively enrolled 220 commercial chicken farms in our study. Face-to-face interviews were conducted using a semi-structured questionnaire with 220 commercial poultry owners for the quantitative component of the study. We performed a priori power analysis using Cohen's *f*^2^ = 0.15 (medium effect) [27] using the 'pwr' package in R. With three predictors, a significance level of 0.05, for achieving 80% the required sample size was 77 farms. Our sample size of 220, the estimated statistical power was approximately 0.99, indicating a high likelihood of detecting meaningful associations. Additionally, in-depth interviews (IDIs) were conducted with 20 selected farm owners for the qualitative component based on the extensiveness and relevance of their responses during the quantitative phase. The researchers continued to include the study participants in qualitative interviews until either data saturation [28] was reached or no new insights or information pertaining to the study objectives were identified. The study included small and medium-sized farm owners of varying ages and sexes, while large farmers and those unwilling to participate were excluded. Commercial poultry farms with fewer than 500 birds were categorized as small-scale, while those with 501–5000 birds were classified as medium scale. Initially, we compiled a list of poultry farms in each area with the assistance of the upazila livestock officer and local farmers. From this list, farms were randomly selected to minimize potential selection bias. Data collectors spent considerable time on each farm to build trust with the farmer, with the average interview lasting approximately 40–50 min.

### 2.3. Study Tool

A semi-structured questionnaire was developed in English by an expert scientific panel comprising two epidemiologists, two veterinary physicians, two anthropologists, and one economist, all with prior experience in poultry farming research. The questionnaire was then translated into Bangla and subsequently back translated into English by different translators to validate the accuracy of the translation. The Bangla version of the questionnaire was then pretested and finalized after necessary modifications. The pretesting was conducted in six (06) small and medium scale commercial poultry farms including equal number (02) of Broiler, Sonali, and layer farms. The questionnaire covered sociodemographic information of the farm owners farm characteristics (e.g., flock size, number of workers, number of sheds, etc.), as well as economic aspects, including questions on the number of batches, profit amounts, and direct-indirect losses during the pre-pandemic, pandemic, and post-pandemic periods.

An IDI guideline was also developed to explore the reasons for losses, the impacts of the lockdown, how farm owners managed COVID-19 challenges, and their recommendations. These interviews were recorded using an audio recorder. Quantitative data from the semi-structured questionnaire were collected by trained research associates, while the IDIs were conducted by anthropologists.

### 2.4. Data Analysis

For quantitative data, Microsoft Excel (Microsoft Office Professional Plus 2021, Microsoft Corporation, Redmond, WA, USA), R Studio version 2022.02.2, [29] and STATA 17.0 (Stata Corp, 4905 Lakeway Drive, College Station, Texas 77845, USA) were used for data entry, cleaning, and analysis. Descriptive statistics on the sociodemography of farm owners, farm characteristics, and economic aspects were analyzed using Pearson's chi-square test. Stepwise regression was conducted to select the best model for predicting the average economic loss per farm due to COVID-19. Model selection was guided by both the akaike information criterion (AIC) and the bayesian information criterion (BIC), balancing model fit and parsimony. The final model with the lowest AIC and BIC values included reduced egg price, reduced bird price, and increased feed price as significant predictors. Given that the dependent variable, loss during the COVID-19 pandemic, was not normally distributed, we applied a log transformation before fitting a linear model. The regression model incorporated variables, such as average reduced egg price, reduced bird price, and increased feed price per farm to estimate the average loss. This model was then used to extrapolate the total national loss by multiplying the average loss per farm by the total number of poultry farms.

For the qualitative data, IDIs were transcribed verbatim and subjected to thematic analysis. Researchers repeatedly reviewed the transcripts, systematically coding them, and identifying new themes. This analysis also incorporated consideration of several a priori themes, such as reasons for economic losses, coping mechanisms, and recommendations [30]. The explanatory interpretation was further validated by cross-referencing with the survey data [31].

### 2.5. Ethical Approval

This study was conducted by following the Declaration of Helsinki and the research protocol was approved by the Ethics Committee of Chattogram Veterinary and Animal Sciences University (Memo no. CVASU/Dir (R&E)EC/2020/241(5)). Informed written consent was obtained from each participant prior to the commencement of interviews. Participants' anonymity and confidentiality were strictly maintained throughout the research process. Furthermore, participants were informed beforehand that the researchers would not provide any financial or other benefits for their participation, nor offer compensation if they were found to be significantly impacted financially by the COVID-19 pandemic.

## 3. Result

### 3.1. Sociodemographic Characteristics

**Figure 1:**
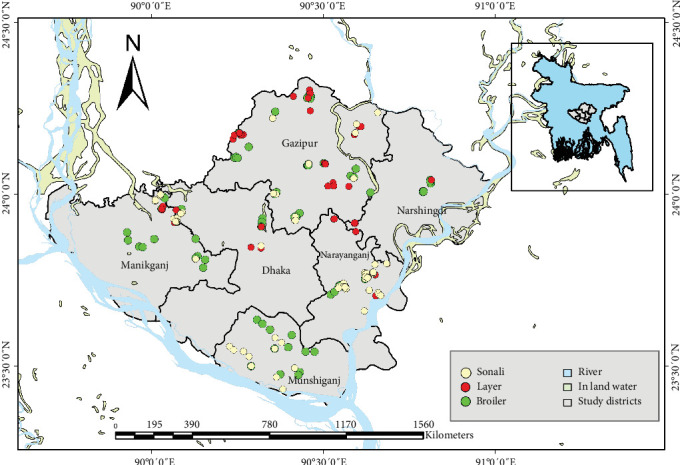
Spatial location of studied farms across different districts in Bangladesh.

Among the 220 study participants, the majority were male (93.18%). Data were collected from six districts: Narshingdi (16 participants, 7.27%), Narayanganj (57 participants, 25.91%), Munshiganj (23 participants, 10.45%), Manikganj (28 participants,12.73%), Gazipur (69 participants, 31.36%), and Dhaka (27 participants, 12.27%) (Figure 1, Table 1). The largest age groups were 18−30 years and 31−40 years, comprising 66.36% of the participants. Over half of the participants (64.55%) were well educated having completed graduation. More than half (58.64%) relied solely on poultry business for their income, while the rest had additional sources of income.

The majority of farm owners had 1–5 years of experience in poultry farming, yet only 16.36% had received any formal training on poultry farming. Most farms (70%) were operated by the owner and their family members, with only 17.73% employing separate workers for farm activities. Given that our study included only small and medium-scale farmers, most (77.27%) farms had only one flock.

Regarding farm types, we identified 78 broiler farms (35.45%), 85 Sonali farms (38.64%), and 57-layer farms (25.91%). There was a significant association (*p* < 0.05) between the type of farm and the likelihood of experiencing financial losses during the COVID-19 pandemic (Table 1).

Supporting Information 1: Table S1 presents the economic loss status of different bird species according to the sociodemographic characteristics of the farm owners. Each demographic variable exhibited substantial economic losses during the COVID-19 pandemic. However, none of the demographic variables, except for the location (district) of Sonali farms, showed a significant relationship with the economic losses incurred during the pandemic.

**Figure 2:**
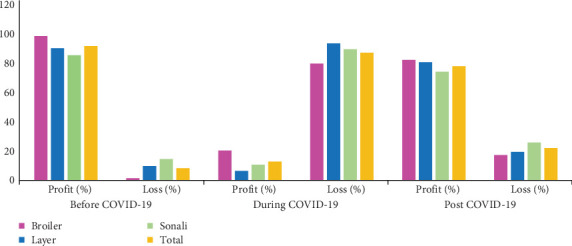
Status of profit or loss before, during, and post-COVID-19 period according to bird species.

Before the pandemic, the majority of farms (91.75%) were profitable, with 98% of broiler farms, 89.47% of layer farms, and 86.11% of Sonali farms reporting profits. However, during the pandemic, overall profitability dropped dramatically, with only 12.27% of farms managed to remain profitable. Specifically, 20.51% of broiler, 8.24% of layer, and 7.02% of Sonali farms remained profitable. In the post-COVID-19 lockdown period, a significant recovery was observed, with 82.05% of broiler farms, 71.76% of layer farms, and 80.7% of Sonali farms reporting profits (Figure 2).

During the pandemic, the mean number of batches raised by broiler poultry farmers decreased by 20.62% (from 7.13 to 5.66). Similarly, the mean duration of layer farming dropped by 5.1% (from 11.96 months to 11.35 months) and the mean number of batches for Sonali poultry farming declined by 16.43% (from 4.87 to 4.07) (Supporting Information 2: Figure S1).

**Figure 3:**
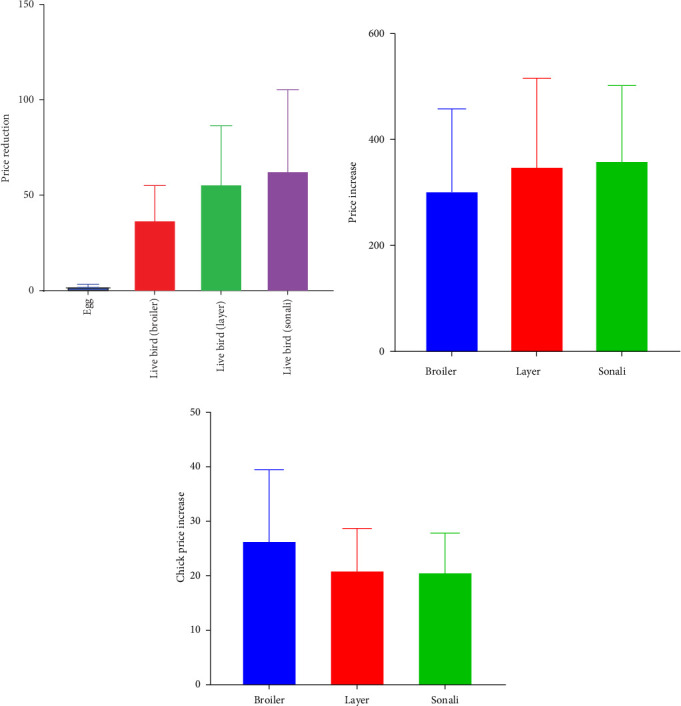
(A) Mean reduction in egg and bird prices during the COVID-19 pandemic in Bangladesh, (B) increase in chick prices during COVID-19, and (C) increase in feed prices during the COVID-19 pandemic.

During the pandemic period, as a result of lockdowns, isolation, and stringent preventive measures enforced by the government and local authorities, the prices of eggs and birds decreased, whereas the prices of feed and chicks increased. Specifically, the average decrease in egg price was 2.53 BDT, while the mean prices for live broiler, layer, and Sonali birds decreased by 37.47, 56.25, and 63.13 BDT, respectively (Figure 3A). Conversely, the feed prices for broiler, layer, and Sonali birds increased by 304.2, 350.62, and 361.73 BDT, respectively (Figure 3B). Additionally, the mean prices of broiler, layer, and Sonali chicks increased by 26.58, 21.14, and 20.82 BDT, respectively (Figure 3C).

**Figure 4:**
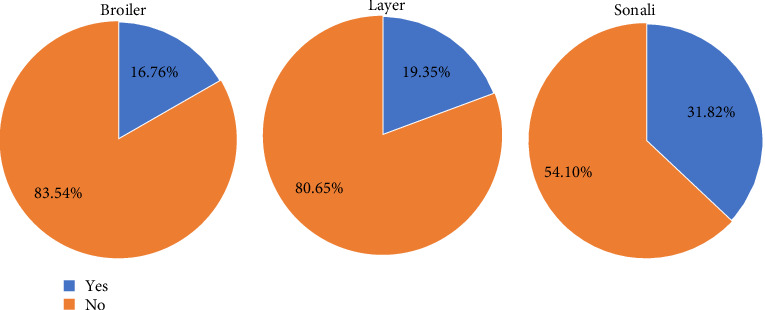
Percentage of farmers who changed their trading pattern/supply chain during the COVID-19 pandemic.

**Figure 5:**
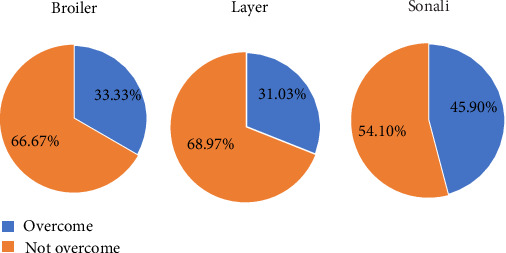
Percentage of farmers who overcame their losses after the COVID-19 pandemic.

During the pandemic period, a significant majority of broiler (83.54%) and layer (80.65%) farmers reported changes in their trading patterns or supply chains, whereas approximately half of the Sonali farmers (54.10%) reported similar changes (Figure 4). After the pandemic period, about 33.33% of broilers, 31.03% of layer, and 45.90% of Sonali farmers managed to recover their losses. However, most farmers indicated during interviews that they had not yet fully overcome the financial impact (Figure 5).

### 3.2. Estimation and Prediction of the Economic loss

We used stepwise regression to identify the best model for predicting the average loss per farm due to COVID-19, selecting the model with the lowest AIC. In univariate regression analysis, the predictors “reduced bird price”, “reduced egg price”, and “increase feed price” were found to be significantly associated with losses incurred during the COVID-19 pandemic (Table 2).

**Figure 6:**
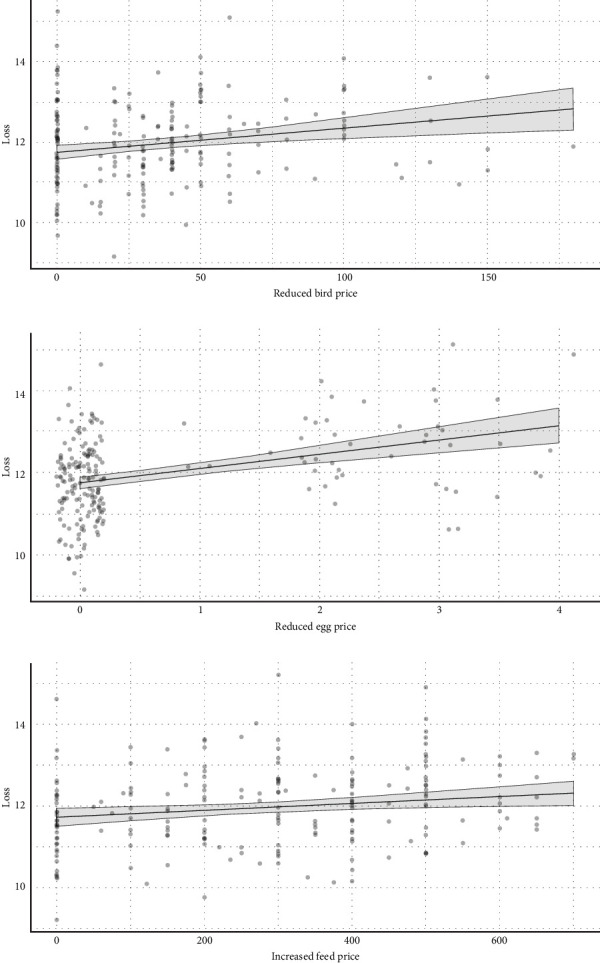
(A–C) Estimated marginal means and their 95% confidence intervals for significant independent variables, adjusted for all other effects.

After adjusting for all other effects, the line graph of estimated marginal means and their 95% confidence intervals predicts a significant inverse relationship between the dependent variable “loss” and the independent variables: decreased egg price, decreased bird price, and increased feed price.  Figure 6A illustrates an ascending curve, revealing that a decrease in each unit of egg price inversely correlates with an increase in economic loss for the farmers (*p*-value = 0.001). In Figure 6B, a decrease in each unit of live bird price shows an increase in economic loss (*p*-value < 0.001).  Figure 6C presents a relatively flat but still ascending curve, indicating that an increase in feed price is associated with an increase in loss during the COVID-19 pandemic (*p*-value = 0.010).

To predict the average loss per farm, we used the average values of reduced egg price, reduced bird price, and increased feed price. The model is as follows:

  $$\begin{array}{c} \text{Average loss}=\exp\left(11.31+0.01\times \text{average reduced bird price}+0.35\times \text{average reduced egg price}+0.0008\times \text{average increased feed price}\right) \end{array}$$

This calculation yields an average loss of 1,52,023.4 BDT ≈ $1407.6

The national loss was then estimated by multiplying the total number of farms by the loss per farm:

  $$\begin{array}{c} \text{National loss}=\text{Total farms}\times \text{loss per farm} \end{array}$$

  $$\begin{array}{c} =70,000\times \text{\$}1407.6=\text{\$}98.5\text{million} \end{array}$$

Thus, the estimated nationwide loss during the COVID-19 pandemic faced by small and medium-scale commercial poultry farms was 98.5 million USD.

This section of the paper systematically explores the impacts of COVID-19 on poultry farming, organized into three major thematic areas: causes of economic losses, coping strategies employed during the pandemic, and recommendations from farmers to mitigate these impacts.

The study initially focused on contextualizing the onset of COVID-19 in Dhaka, Bangladesh, where following the first detected case, the government of Bangladesh implemented several measures, including a nationwide lockdown and repeated enforcement of social distancing, isolation, and movement restrictions across seven distinct phases. During this period, many businesses, particularly small and medium-scale enterprises like poultry farming, faced significant financial challenges. The poultry farmers reported considerable economic losses primarily due to decreased consumer demand and increased operational costs of farming during the pandemic.

### 3.3. Dwindling Consumer Demand

During the pandemic period, stringent measures, such as lockdowns, social distancing, isolation, and other restrictions were implemented to curb the spread of COVID-19 cases, resulting in widespread closures of offices, industries, and garments sectors, which significantly impacted consumer behavior. The enforcement of these measures led to a sharp decline in public gatherings, such as weddings and other events, further compounded by restrictions on hotels, restaurants, food shops, and local marketplaces, which were only allowed to operate on limited days. Consequently, these restrictions severely reduced the customer base for local bazaars or markets, leading to a notable decrease in poultry consumption, as uniformly reported by all respondents. This decline is exemplified by the experiences of a small-scale poultry farmers, who reported a significant reduction in demand for his Sonali chickens during this period. The below example as described the situation by a small-scale poultry farmer who raised Sonali birds underscores the broader economic impact of the pandemic on small poultry enterprises:

*“There was no demand for chickens. Restaurants were closed and social events or gatherings were nonexistent. Consequently, I had to sell chickens at a diminished price with loss.”* (*IDI 14*)

The study found that farmers typically sold their poultry locally to dealers, retailers, and vendors who then transported and sold the poultry in Dhaka, the capital city. However, the implementation of strict movement restriction policy like lockdowns, social isolation, and general leaves, severely hampered poultry trading during the COVID-19 pandemic. Farmers reported that most dealers and retailers closed their shops during the lockdowns, and vendors only operated their shops on specific days as permitted by local government or authorities.

*“After corona, our farm was closed for 6 months. All the local markets, restaurants were closed, and continued lockdown in Dhaka. There were no wedding ceremonies, so where would I sell the poultry? So, that time I didn't raise any poultries. 300–350 chickens were ready for sell that week, and there was the lockdown so couldn't sell them.”* (Sonali poultry farmer, Narayanganj)

The farmer explained the severe impact of the COVID-19 pandemic on his poultry business. When the lockdown was enforced, all local markets and restaurants were closed, including those in Dhaka, the capital city. This closure meant that there were no outlets for selling his chickens. Additionally, wedding programs, which are a major part of the food market and typically include chicken meat as a key item on the menu, were not taking place. With these major sales channels unavailable, the farmer faced a critical situation. He had chickens ready for sale, but due to the lockdown, he couldn't sell them. Consequently, he decided to halt poultry raising entirely during the 6-month shutdown.

### 3.4. Rumors and Socio-Behavioral Shifts in Poultry Consumption

During the initial stages of the pandemic, widespread uncertainty about the cause and origins of COVID-19 fueled the spread of various rumors. Mainstream media outlets sometimes broadcast these rumors to attract viewers, while social media platforms became hotbeds for amplified unverified information. Few respondents in the study also reported rumors that incorrectly blamed poultry for transmitting COVID-19 to humans. Misconceptions that consuming poultry consumption and its products could lead to COVID-19 infections were prevalent until subsequent rumors suggested bats as potential reservoirs of the virus. One broiler farmer, who lost two immediate family members to COVID-19, reported that the public was extremely anxious and scared during this period. He observed a marked decline in poultry consumption driven by these fears. He stated,

*“People doubted poultry consumption as it may spread corona. We were under a lot of pressure at that time… people didn't eat chickens at all, it took a lot of time to sell those chickens.”* (*IDI 18*)

During the pandemic, not only consumers but also poultry farmers experienced significant sociobehavioral changes, influenced heavily by widespread rumors. These rumors instilled fear among farmers about the possibility of contracting the virus from their poultry. Such fears led many of them to shut down their farm during the pandemic period. One of the respondents, who owned a broiler farm, stated,

*“I didn't know if I could stay alive or not during corona. I was sad seeing the situation of many other countries, so I stopped raising for 3 months. Then I saw nobody was following the rules so I started raising chickens again.”* (*IDI 1*)

A few farmers reported observing unusual poultry deaths during COVID-19, which they speculated might be linked to the COVID-19. These speculated correlations caused panic among farmers, especially as COVID-19 cases surged in their villages or communities. The spread of rumors and fear among the villagers compounded the situation, leading to a temporary shutdown of their farms. This closure had a profound impact on their livelihoods and cast a shadow of uncertainty over their future prospects. One of the Sonali poultry farmers stated the dire situation as below:

*“Many poultry died but no disease could be detected, I cut the birds to diagnose the disease of the poultry, still nothing was detected and even the village doctors couldn't diagnose”* (*IDI 13*).

### 3.5. Augmented Outlays for Poultry Operations During COVID-19

Farmers complained significant increases in operational costs of poultry farming, which placed considerable strain on their profitability and overall farming operations. They reported that the price of all essential farming inputs, such as poultry feed, chicks, medicines, and litter/husk raised significantly. Notably, the soaring expenses of poultry feed, which constitutes a substantial portion of their farming budget, became a major concern for the majority of farmers. Moreover, the heightened costs for chicks and medicines further amplified their financial burdens. The rising price of litter and husks, essential for maintaining sanitation and proper hygiene, added to these challenges. Consequently, a multitude of farmers voiced their frustration and concern over the sustainability of their poultry enterprises amidst the mounting financial challenges.

According to the farmers, poultry feed and medicine industries heavily rely on imported raw materials. The study noted a notable decline in the production of poultry feed and medicines, attributed to cross-border restrictions imposed during the pandemic. Additionally, disruptions in transportation further compounded the situation, resulting in escalated farming costs throughout the pandemic period. Our study respondents frequently highlighted the significant impact of these transportation issues on the supply chain, particularly affecting feed availability and the distribution of poultry products. These challenges were repeatedly emphasized by the farmers as significant barriers to their farming operations. In response to this topic, a female owner of a broiler farm provided the following feedback:

*“During COVID-19, all the transport and vehicles were unavailable so I had to stop farming for 5–6 months, besides, I couldn't sell chickens, and I had to use feed, medicine (with the increased price the loss was around one lac taka. If I didn't stop farming, then I could make a profit of 4 lac taka.”* (*IDI 10*)

### 3.6. Disruption of the Supply Chain and Selling Networks

The augmentation of farming costs also contributed significantly to the disruption of traditional supply chains and selling patterns during the pandemic.

Farmers reported that the majority of dealers, middlemen, and suppliers had to close their businesses due to COVID-19 restrictions. Consequently, they experienced disruptions to the established supply chain and selling networks they had previously relied upon. Before the pandemic, it was common practice for farmers to buy DOCs from dealers or hatcheries, and to source feed and husk from same or different dealers. In addition, some dealers also engaged in veterinary medicine businesses and provided assistance for treating poultry when required.

In the poultry business in Bangladesh, a credit system is commonly used, where dealers provide credit to farmers for DOCs, feed, and other necessary supplies. Farmers are expected to repay these credits after selling their poultry or poultry products. Interestingly, the same dealers who extend credit typically also purchase live birds or eggs from the farmers. This arrangement allows dealers to earn profit without taking on much of the associated risks.

However, during the pandemic, dealers stopped providing credit to farmers and began demanding repayment of existing debts. This shift placed additional financial pressure on the farmers, significantly compounding their difficulties during an already challenging period. A farmer who raised broiler poultry expressed how these changes affected his operations and financial stability as follows:

*“During the lockdown*, *I had a loss because the dealer didn't give feed*, *as the factory was closed*, *production was decreased.”* (*IDI 18*)

Similarly, another farmer, who raised Sonali poultry, also shared insights on the same issue,

*“During COVID-19, I couldn't sell the poultry as the wholesaler wasn't there to buy it, all shops and markets were closed so I had to sell the chickens to the local neighborhood in due. I have now recovered from the due payment. I thinks I couldn't overcome this loss yet.”* (*IDI 6*)

**Figure 7:**
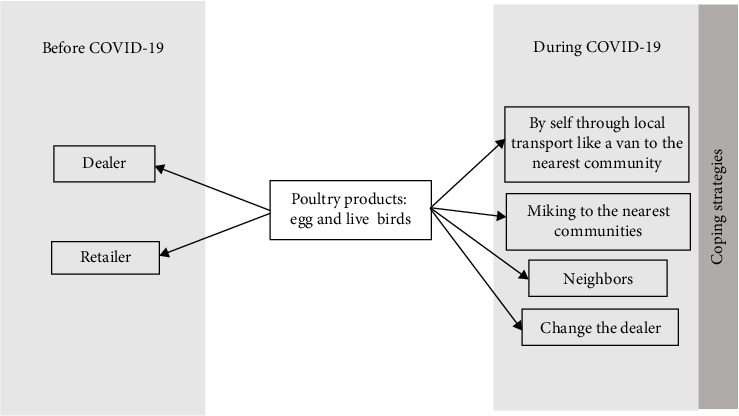
Change in the selling pattern during the COVID-19 pandemic.

During the lockdown, the usual patterns of selling were significantly disrupted as regular retailers or dealers became irregular or discontinued their businesses altogether. This unforeseen setback severely impacted farmers, as the poultry continued to age without gaining additional weight, thereby increasing the costs of maintenance without corresponding revenue. The increased burden forced farmers to change their selling strategies and cope with a neo-normal situation (Figure 7).

### 3.7. Coping Strategies During COVID-19

Throughout the pandemic, small and medium-scale commercial poultry farmers faced significant challenges, but devised some coping strategies to mitigate the impacts. While a few respondents expressed a sense of helplessness, with no other option than to close their farms, others adapted by changing their supply chains and selling patterns. To overcome financial losses, some of the farmers resorted to borrowing from banks, non-governmental organizations (NGOs), relatives, or other sources. Some of them even liquidated assets, such as cattle and cropland, and with one respondent reported closing other businesses to run his poultry farm during the crisis. Although, some of the respondents received government incentives intended to offset their losses, the aid was often insufficient to fully recover the burden of economic catastrophes.

#### 3.7.1. Altered Selling Strategies

In response to pandemic-induced closures of dealerships and retail outlets, poultry farmers had to significantly alter their selling strategies. As depicted in Figure 7, farmers adopted diverse strategies to cope with the restrictions to sell eggs and live birds through traditional channels. Facing critical situations where mature birds could not be sold through regular systems, farmers often improvised their selling options by utilizing local transport, such as vans to reach nearby consumer communities, announcing sales through miking to the community, or selling directly to their neighbors, and, in fortunate cases lucky, securing new dealers.

Some farmers recalled their stressful experiences of unsold poultry piling up day by day, necessitating continued expenditure on costly feed, which compounded their financial losses. What was once considered an asset had become a substantial financial burden. Consequently, they were compelled to adopt unconventional and diverse strategies to sell their birds. Specific response from one farmer on this issue stated below-

*“I had to sell eggs by going to people's houses, I had to walk and sell.”* (*IDI 20*, *A layer farmer from Manikganj*)

#### 3.7.2. Borrowed Funds and Liquidated Assets

During the COVID-19 pandemic, poultry farmers faced significant financial challenges that compelled them to seek various funding options to sustain their poultry businesses. To manage the severe losses affecting most of their poultry batches, many respondents obtained loans from banks, NGOs, loan sharks, relatives, and even relatives or neighbors. For those unable to secure loans, asset liquidation became necessary, leading to the sale of valuable possessions, like crop fields and domestic cattle. One of our respondents reported having to shut down another business to maintain poultry farming. Another farmer mentioned becoming entangled in a burdensome cycle of debt with a dealer, which consumed a significant portion of his profits, further complicating his financial situation. Additional comments from the respondents on these financial strategies are stated below,

*“To overcome this loss, we took a loan from an NGO. I was in debt of taka 438000 takas to our dealer but now the amount has been reduced to 176000 takas.”* (*IDI 7*)

*“I didn't take any bank loan but had to sell our land/property. I had a tough time during the lockdown as there was a transport problem, and financial problems, also my husband lost his job. The government can help in a lot of ways during this tough time.”* (*IDI 10*)

#### 3.7.3. Stimulus Packages From Government

In the post-pandemic period, the Government of Bangladesh implemented financial support measures for poultry farmers, although not all poultry farmers were included for these stimulus packages. The financial aid provided was intended to alleviate some of the economic hurdles faced by the farmers, yet most of the respondents who received financial incentives reported that the amount of the assistance was insufficient to fully mitigate their losses. One of the farmers who received government incentives stated,

*“To overcome my loss, I supposed to get a loan but couldn't. But a few days ago, I received a govt. grant of 17,100 taka, but my loss was two lacs.”* (*IDI 3*)

### 3.8. Lessons Learned by Farmers From COVID-19 and Their Recommendations

**Figure 8:**
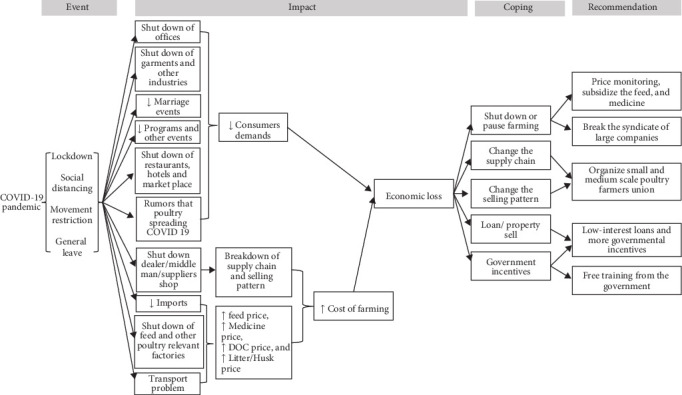
Graphical summary of the explorative findings from small and medium-scale commercial poultry farmers.

During the COVID-19 pandemic, poultry farmers gained valuable insights into managing their operations under crisis conditions. They shared these lessons and recommendations for mitigating financial losses and better preparing for future pandemics (Figure 8). One key suggestion was the implementation of government or NGO-supported free training programs for small and medium-scale commercial poultry farmers. These training programs would focus on essential skills, such as financial management, feeding, and treatment practices, aiming to empower farmers with the knowledge to make informed decisions independently, rather than relying on neighboring farmers and dealers. In addition, the farmers advocated for increased governmental incentives, noting that existing supports were inadequate for sustaining their livelihoods. Furthermore, they also highlighted the importance of accessing low-interest loans from banks, which could provide critical financial relief, particularly for small business operators like themselves.

The farmers also recommended the inclusion of all small- and medium-scale commercial poultry farmers in the existing poultry farmers' association to better elevate their concerns and address their demands effectively. This inclusion would enable them to collectively raise their voices confronting the monopolistic practices of large companies, which often oppress small farmers to dominate the entire market. They also proposed that the government should implement policies to support small-scale farmers and exert pressure on these companies to ensure fair practices. Additionally, most participants suggested that the government should establish a price monitoring system and, if feasible, subsidize the cost of poultry feed and medicine to support the sustainability of small- and medium-scale operations. Additional comments from respondents are outlined below:

*“Government can help us financially. Government can also decrease the feed price because the price is too much now. Also, farm training can be given to us from the Upazila (subdistrict) livestock department. We are doing everything on our own, we didn't get any training regarding farming.”* (*IDI 10*, *Broiler farmer*)

*“Some large poultry industries are controlling the whole business and benefited. They have lacs of poultry. When they want to sell their poultries, the market price is high, 150–170 taka per kilogram and when they finished their sells, the market price gets down to 140 taka. These need to stop. The government needs to take action for this.”* (*IDI 16*)

## 4. Discussion

Small and medium-scale poultry farms are the primary source of cost-friendly animal protein for low- and middle-income communities. In countries like Bangladesh, the consumption of eggs and chicken meat plays a vital role in meeting the nutritional needs of the population [32, 33]. Unfortunately, the recent COVID-19 pandemic has had detrimental effects on public health and various economic sectors, including the poultry industry [34]. As a consequence, small- and medium-sized poultry farms in Bangladesh have experienced significant economic setbacks, with many still struggling to recover from the pandemic's impacts. The persistent increases in the prices of feed, chicks, medicine, and vaccines continue to pose challenges for these farms, complicating their recovery and ongoing farming operations. In March 2020, the closure of hotels, restaurants, bakeries, and fast-food establishments as a preventive measure to stop the spread of COVID-19 has significantly reduced the demand for poultry meat and eggs. Around 70% of poultry farms had temporarily ceased operations [35]. At the onset of the COVID-19 pandemic, widespread misinformation and fear linked the consumption of chicken meat and eggs to the transmission of the virus among humans [36, 37]. This situation posed a severe threat to the livelihoods of millions of small-scale poultry farmers in Bangladesh [37]. Any disruption in the poultry supply chain can lead to significant economic consequences, as witnessed during the pandemic. The credit system where distributors or dealers extended credit to small-scale farmers was a critical support mechanism during the pandemic. However, this system was significantly disrupted during the COVID-19 period, leading to severe financial distress among many farmers.

Prior to this study, there was a lack of systematic estimation of the total economic losses experienced by small- and medium-scale commercial poultry farms across Bangladesh. This research aims to fill this gap by providing evidence for researchers, policymakers, and other stakeholders within the poultry industry. Our findings reveal that these farms incurred substantial losses amounting to approximately 98.5 million USD during the COVID-19 pandemic, which is a significant impact for a country like Bangladesh, where the poultry sector involves approximately 70,000 small- and medium-scale commercial farms, with a total investment of 35,000 crore BDT (~3 billion USD) in the poultry sector and sustains around 6.0 million livelihoods [38]. Previous studies have indicated a 35% decrease in the production of DOCs, eggs, and live birds during the pandemic [39], with selling prices for eggs and live birds often falling below production costs, exacerbating economic challenges faced by the sector. The Bangladesh Poultry Industries Central Council reports that the entire poultry industry in Bangladesh, including feed, hatchery, and associated sectors, incurred an estimated loss of 7000 crore BDT (~642.80 million USD)[40, 41]. Our findings are consistent with these findings and reports, underscoring the extensive financial challenges faced by the sector during this period. This study reported changes in trading patterns or supply chains among significant majority of broiler (83.54%), layer (80.65%) farmers, and approximately half of the Sonali farmers (54.10%) which is aligned with some previous studies also [20]. This information exposes the necessity of special attention to preserve the usual supply chain in poultry industries during any epidemic or pandemic.

**Figure 9:**
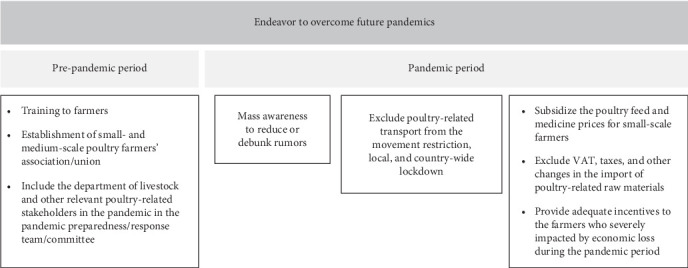
Summary of the endeavors needs to be modified to address the potential future epidemics and pandemics.

Poultry farmers impacted by the COVID-19 pandemic endeavored to adapt to the evolving circumstances. A significant proportion of these farmers experienced severe economic hardships due to the pandemic's impact. While, some were able to sustain their farming operations through government incentives or by securing loans, some found it unsustainable to continue and opted to pause their farming activities during the period of restrictions. This pattern of impact and response is consistent with findings from previous studies conducted in Bangladesh and other low-income countries [3, 18, 42, 43]. This study delves into the perspectives of poultry farmers on existing remedies and future policies aimed at recovering the economic catastrophes wrought by the COVID-19 pandemic on the poultry industry. Unlike previous research that predominantly drew on scientists' observations and perceptions to formulate recommendations, this research brings to light new viewpoints from farm owners themselves. Some of the novel insights from these farmers include advocating for the formation of a trade union for small- and medium-scale poultry farmers, dismantling the monopoly of larger farms, and proposing subsidies for feed, vaccines, and medicines to support small poultry farmers. Farmers' additional recommendations are consistent with those documented in a few previous articles. They emphasize the necessity for government authorities to monitor prices, provide training to farmers, remove broker systems, and third parties from the supply chain, and offer low-interest loans and incentives as part of government support measures (Figure 9) [3, 38].

The study is subject to certain limitations as it was not possible to collect representative data from all divisions and geographic regions of Bangladesh, despite efforts to ensure an adequate sample size. Consequently, the findings may not be broadly generalizable. Additionally, the qualitative data did not cover all regions or cultural dimensions of Bangladesh, although it still provides an important snapshot of the situation. There is a potential overpresentation of the financial scenario, which may stem from respondents' biases towards demand characteristics. To counteract this bias, researchers clarified to respondents that participation in the interviews would not yield any compensatory incentives for their losses and that they were not affiliated with any government bodies. Moreover, recall bias related to counts and numbers was addressed by interviewers cross-verifying the size and number of flocks, and sometimes confirming information with family members regarding farm shutdowns, gap months, and flock sizes to ensure the accuracy of the responses. Farms with major discrepancies between reported and actual conditions were excluded from the study. Despite these limitations, the study also possesses several strengths. Notably, it included all types of poultry farms, encompassing various species across six districts. The study's major strength lies in its detailed capture of farmers' perspectives and insights, which are invaluable for informing future strategies in pandemic preparedness.

## 5. Conclusion and Recommendations

The marginal and small-scale poultry sectors in Bangladesh experienced significant impacts due to the COVID-19 pandemic and subsequent lockdowns, with many farmers still facing challenges in recuperating their financial losses. It is imperative for the government to extend support to these farmers, enabling them to continue contributing to protein supply and poverty alleviation within the community. Recognizing and addressing the challenges encountered by small- and medium-scale poultry farmers during the COVID-19 pandemic and subsequent lockdowns is crucial in preparing for future pandemics or disease outbreaks. Further research is recommended to explore coping mechanisms and strategies to minimize economic losses, enhancing resilience in these critical sectors. A comparative study between poultry farmers who adopted different business models (e.g., cooperative-based versus independent farming) can provide deeper insights into resilience strategies.

In Bangladesh, a significant number of small and medium-scale commercial poultry farmers lack formal training in poultry farming and management, with many farm owners and workers relying on self-taught methods [44–46]. This gap in formal education often leaves them vulnerable to disruptions in the normal supply chain, changes in market dynamics, and disease outbreaks, which introduce new challenges and complicate their operations. To address these issues, it is crucial to provide a comprehensive training program specifically tailored for these small- and medium-scale farmers. Such training should cover effective management practices during outbreaks or pandemic situations effectively. Given the economic vulnerabilities faced by many farm owners, offering this training free of charge through governmental organizations or NGOs would be beneficial. Furthermore, introducing a requirement for mandatory training as a prerequisite for obtaining a farming license could significantly enhance preparedness and resilience among poultry farmers in future pandemics.

The absence of a centralized union for small- and medium-scale poultry farmers makes it challenging for them to effectively communicate their concerns to policymakers and government agencies. Issues involving dealers, feed manufacturers, large companies, hatcheries, or government authorities often remain unresolved due to this absence of representation. Establishing farmer unions could provide a platform for these farmers to advocate for their rights and negotiate for benefits, including financial incentives from the government, particularly during periods of crisis like the pandemic. The committee responsible for pandemic preparedness and formulating relevant policies and strategies should engage in multilevel stakeholder collaborations, including with the Department of Livestock and other entities involved in the poultry industry. Involving these stakeholders will bring valuable insights from the perspective of micro businesses and aid in ensuring the sustainability of the poultry sector, a significant source of protein for the nation. Additionally, the committee could recommend that transport related to the poultry industry be exempt from movement restrictions or lockdowns, thereby minimizing disruptions to this essential food supply chain.

In situations where information is scarce, rumors often proliferate, as observed in the poultry industry in Bangladesh during the pandemic. To address this issue and prevent its recurrence in future pandemics, it is imperative for the government to proactively disseminate accurate, up-to-date and timely information to the public. This can be achieved through comprehensive mass awareness campaigns utilizing various channels, such as television, social media, newspapers, and local government communications. Such initiatives will help to effectively curb the spread of misinformation and rumors. Furthermore, in order to ensure the sustainability of small- and medium-scale poultry farms and enhance their competitive edge against larger enterprises, government support should be prioritized. The government should offer subsidies, additional incentives, and a temporary tax relief to small and medium-scale farmers to offset losses incurred during pandemic. Additionally, ensuring access to low-interest loans is also essential for these farmers. Implementing these measures will help sustain small poultry businesses, which are integral to the economic backbone of rural communities in Bangladesh, alongside crop production.

## Figures and Tables

**Table 1:** Sociodemographic characteristics of study participants (*N* = 220).

Variable	Frequencies	Percentages	*χ* ^2^
District			6.06
Narshingdi	16	7.27	
Narayanganj	57	25.91	
Munshiganj	23	10.45	
Manikganj	28	12.73	
Gazipur	69	31.36	
Dhaka	27	12.27	
Age			1.71
18–30	73	33.18	
31–40	73	33.18	
41–50	54	24.55	
>50	20	9.09	
Gender			0.89
Male	205	93.18	
Female	15	6.82	
Educational status			2.45
Illiterate/primary	24	10.91	
Secondary/higher secondary	54	24.55	
Graduated and above	142	64.55	
Occupation			0.24
Only poultry	129	58.64	
Poultry with other	91	41.36	
Experience			0.79
1–5	93	42.27	
>5–10	58	26.36	
>10	69	31.36	
Training			0.1044
Yes	36	16.36	
No	184	83.64	
Who works at the farm			3.66
Owner and family member	154	70.00	
Worker	28	12.73	
Both	38	17.27	
Type of Bird			7.67^*∗*^
Broiler	78	35.45	
Sonali	85	38.64	
Layer	57	25.91	
Flock size			0.31
1	170	77.27	
>1	50	22.73	

*Note: χ* ^2^, Chi-square value.

*⁣* ^*∗*^Significant at 5% (*p* < 0.05),

**Table 2:** Estimates with 95% CI and *p*-value of the linear model.

Predictors	Loss		
	Estimates	CI	*p*
Intercept	11.31	11.07–11.56	**<0.001**
Reduced bird price	0.01	0.00–0.01	**0.001**
Reduced egg price	0.35	0.23–0.46	**<0.001**
Increased feed price	0.0008	0.00–0.00	**0.010**
Observations	202		

## Data Availability

The data that support the findings of this study are available from the corresponding author upon reasonable request.

## Supporting Information

Supporting Information 1 Table S1: Economic loss status of Broiler, Sonali, and layer farms by sociodemographics of farm owners and farm characteristics. The table presents the economic loss status of different bird species according to the sociodemographic characteristics of the farm owners. Each demographic variable exhibited substantial economic losses during the COVID-19 pandemic. However, none of the demographic variables, except for the location (district) of Sonali farms, showed a significant relationship with the economic losses incurred during the pandemic.

Supporting Information 2 Figure S1: Mean number of batches/months of farming per year before, during, and after the COVID-19 pandemic. During the pandemic, the mean number of batches raised by broiler poultry farmers decreased by 20.62% (from 7.13 to 5.66). Similarly, the mean duration of layer farming dropped by 5.1% (from11.96 months to 11.35 months), and the mean number of batches for Sonali poultry farming declined by 16.43% (from 4.87 to 4.07).
