# Supplementary material for: Estimating Economic Losses in Commercial Chicken Farms During COVID-19 Pandemic in Bangladesh: Lessons Learned for Future Pandemic
Source: Transbound Emerg Dis. 2025 Jun 22;2025:4935897. doi: 10.1155/tbed/4935897 (PMC12206572; doi:10.1155/tbed/4935897)
Supplement: Supporting Information 1 — Table S1: Economic loss status of Broiler, Sonali, and layer farms by sociodemographics of farm owners and farm characteristics. The table presents the economic loss status of different bird species according to the sociodemographic characteristics of the farm owners. Each demographic variable exhibited substantial economic losses during the COVID-19 pandemic. However, none of the demographic variables, except for the location (district) of Sonali farms, showed a significant relationship with the economic losses incurred during the pandemic. [file 4935897.f1.docx]

**Table S1: Economic loss status of Broiler, Sonali, and layer farms by socio-demographics of farm owners and farm characteristics:**

|  | **Broiler**  (n=78) | | | | **Sonali**  (n=85) | | | | **Layer**  (n=57) | | | | **Total**  (n=220) | | | |
| --- | --- | --- | --- | --- | --- | --- | --- | --- | --- | --- | --- | --- | --- | --- | --- | --- |
|  | Total loss | Total count | Binomial Probability (95%CI) | P-value | Total loss | Total count | Binomial Probability (95%CI) | P-value | Total loss | Total count | Binomial Probability (95%CI) | P-value | Total loss | Total count | Binomial Probability (95%CI) | P-value |
| **District** |  |  |  |  |  |  |  |  |  |  |  |  |  |  |  |  |
| Narshingdi | 10(90.91) | 11 | .91(.59-1.0) | 0.99 | 0 | 0 |  | 0.038 | 5(100) | 5 | 1 (.48-1*) | 0.328 | 15 (93.75) | 16 | .94 (.70-1.00) | 0.247 |
| Narayanganj | 6(85.71) | 7 | .86(.42-1.0) |  | 42(97.67) | 43 | .98(.88-.99) |  | 7(100) | 7 | 1(.59-1*) |  | 55 (96.49) | 57 | .96 (.88-.99) |  |
| Munshiganj | 10(83.33) | 12 | .83(.52-.98) |  | 11(100) | 11 | 1(.72-1*) |  | 0 | 0 |  |  | 21 (91.30) | 23 | .91 (.72-.99) |  |
| Manikganj | 11(84.62) | 13 | .85(.55-.98) |  | 6(75.00) | 8 | .75(.35-.97) |  | 7(100) | 7 | 1(.59-1*) |  | 24 (85.71) | 28 | .86 (.67-.96) |  |
| Gazipur | 26(89.66) | 29 | .90(.73-.98) |  | 11(100) | 11 | 1(.72-1*) |  | 27(93.10) | 29 | .93(.77-.99) |  | 64 (92.75) | 69 | .93 (.84-.98) |  |
| Dhaka | 5(83.33) | 6 | .83(.36-1.0) |  | 10(83.33) | 12 | .83(.52-.98) |  | 7(77.78) | 9 | .78 (.40-.97) |  | 22 (81.52) | 27 | .81 (.62-.94) |  |
| **Age** |  |  |  |  |  |  |  |  |  |  |  |  |  |  |  |  |
| 18-30 | 23(85.19) | 27 | .85(.66-.96) | 0.88 | 28(100) | 28 | 1(.88-1*) | 0.164 | 17(94.44) | 18 | .94 (.73-1.0) | 0.077 | 68(93.15) | 73 | .93(.85-.98) | 0.589 |
| 31-40 | 21(87.50) | 24 | .88(.68-.97) |  | 25(92.59) | 27 | .93(.76-.99) |  | 22(100) | 22 | 1(.85-1*) |  | 68(93.15) | 73 | .93(.85-.98) |  |
| 41-50 | 20(86.96) | 23 | .87(.66-.97) |  | 18(85.71) | 21 | .86(.64-.97) |  | 9(90) | 10 | .90(.55-1.0) |  | 47(87.04) | 54 | .87(.75-.95) |  |
| >50 | 4(100) | 4 | 1(.40-1*) |  | 9(100) | 9 | 1(.66-1*) |  | 5(71.43) | 7 | .71(.29-.96) |  | 18(90.00) | 20 | .90(.68-.99) |  |
| **Gender** |  |  |  |  |  |  |  |  |  |  |  |  |  |  |  |  |
| Male | 61(88.41) | 69 | .88(.78-.95) | 0.37 | 76(95.00) | 80 | .95(.88-.99) | 0.167 | 52(92.86) | 56 | .93(.83-.98) | 0.782 | 189(92.2) | 205 | .92(.88-.95) | 0.105 |
| Female | 7(77.78) | 9 | .78(.40-.97) |  | 4(80.00) | 5 | .80(.28-.99) |  | 1(100) | 1 | 1(.025-1*) |  | 12(80.00) | 15 | .80(.52-.96) |  |
| **Educational Status** |  |  |  |  |  |  |  |  |  |  |  |  |  |  |  |  |
| Illiterate/primary | 4(100) | 4 | 1(.40-1*) | 0.37 | 12(100) | 12 | 1(.74-1*) | 0.148 | 8(100) | 8 | 1(.63-1*) | 0.069 | 24(100) | 24 | 1(.86-1*) | 0.169 |
| Secondary/higher secondary | 17(94.44) | 18 | .94(.73-1.0) |  | 18(85.71) | 21 | .86(.64-.97) |  | 12(80) | 15 | .8(.52-.96) |  | 47(87.04) | 54 | .87(.75-.95) |  |
| Graduated & Above | 47(83.93) | 56 | .05(.72-.92) |  | 50(96.12) | 52 | .96(.87-.99) |  | 33(97.06) | 34 | .97(.85-1.0) |  | 130(91.55) | 142 | .92(.86-.96) |  |
| **Occupation** |  |  |  |  |  |  |  |  |  |  |  |  |  |  |  |  |
| Only poultry | 38(86.36) | 44 | .86(.73-.95) | 0.81 | 51(94.44) | 54 | .94(.85-.99) | 0.866 | 30(96.77) | 31 | .97(.83-1.0) | 0.221 | 119(92.25) | 129 | .92(.86-.96) | 0.578 |
| Poultry with other | 30(88.24) | 34 | .88(.73-.97) |  | 29(93.55) | 31 | .94(.79-.99) |  | 23(88.46) | 26 | .88(.70-.98) |  | 82(90.11) | 91 | .90(.82-.95) |  |
| **Experience** |  |  |  |  |  |  |  |  |  |  |  |  |  |  |  |  |
| 1-5 | 26(86.67) | 30 | .87(.69-.96) | 0.90 | 44(91.67) | 48 | .92(.80-.98) | 0.399 | 15(100) | 15 | 1(.78-1*) | 0.461 | 85(91.4) | 93 | .91(.84-.96) | 0.83 |
| >5-10 | 17(85) | 20 | .85(.62-.97) |  | 15(93.75) | 16 | .94(.70-.99) |  | 20(90.91) | 22 | .91(.71-.99) |  | 52(89.66) | 58 | .90(.79-.96) |  |
| >10 | 25(89.29) | 28 | .89(.72-.98) |  | 21(100) | 21 | 1(.84-1*) |  | 18(90) | 20 | .9(.68-.99) |  | 64(92.75) | 69 | .93(.84-.98) |  |
| **Training** |  |  |  |  |  |  |  |  |  |  |  |  |  |  |  |  |
| Yes | 14(87.50) | 16 | .88(.62-.98) | 0.97 | 12(100) | 12 | 1(.74-1*) | 0.350 | 7(87.50) | 8 | .88(.47-1.0) | 0.513 | 33(91.67) | 36 | .92(.78-.98) | 0.944 |
| No | 54(87.10) | 62 | .87(.76-.94) |  | 68(93.15) | 73 | .93(.85-.98) |  | 46(93.88) | 49 | .94(.83-.99) |  | 168(91.30) | 184 | .91(.86-.95) |  |
| **Who work at the farm** |  |  |  |  |  |  |  |  |  |  |  |  |  |  |  |  |
| Owner & family member | 52(85.25) | 61 | .85(.74-.93) | 0.496 | 62(93.94) | 66 | .94(.85-.98) | 0.702 | 24(88.89) | 27 | .89(.71-.98) | 0.353 | 138(89.61) | 154 | .90(.84-.94) | 0.357 |
| Worker | 8(100) | 8 | 1(.63-1*) |  | 8(100) | 8 | 1(.63-1*) |  | 11(91.67) | 12 | .92(.62-1.0) |  | 27(96.43) | 28 | .96(.82-1.0) |  |
| Both | 8 (88.89) | 9 | .89(.52-.1.0) |  | 10(90.91) | 11 | .91(.59-1.0) |  | 18(100) | 18 | 1(.81-1*) |  | 36(94.74) | 38 | .95(.82-.99) |  |
| **Flock Size** |  |  |  |  |  |  |  |  |  |  |  |  |  |  |  |  |
| 1 | 53(88.33) | 60 | .88(.77-.95) | 0.58 | 66(92.96) | 71 | .93(.84-.98) | 0.306 | 35(89.74) | 39 | .90(.76-97) | 0.159 | 154(90.59) | 170 | .91(.85-.96) | 0.450 |
| >1 | 15(83.33) | 18 | .83(.59-.96) |  | 14(100) | 14 | 1(.77-1*) |  | 18(100) | 18 | 1(.81-1*) |  | 47(94) | 50 | .94(.83-.99) |  |

********one-sided, 97.5% confidence interval*
