# Supplementary material for: Estimating Economic Losses in Commercial Chicken Farms During COVID-19 Pandemic in Bangladesh: Lessons Learned for Future Pandemic
Source: Transbound Emerg Dis. 2025 Jun 22;2025:4935897. doi: 10.1155/tbed/4935897 (PMC12206572; doi:10.1155/tbed/4935897)
Supplement: Supporting Information 2 — Figure S1: Mean number of batches/months of farming per year before, during, and after the COVID-19 pandemic. During the pandemic, the mean number of batches raised by broiler poultry farmers decreased by 20.62% (from 7.13 to 5.66). Similarly, the mean duration of layer farming dropped by 5.1% (from11.96 months to 11.35 months), and the mean number of batches for Sonali poultry farming declined by 16.43% (from 4.87 to 4.07). [file 4935897.f2.docx]

*
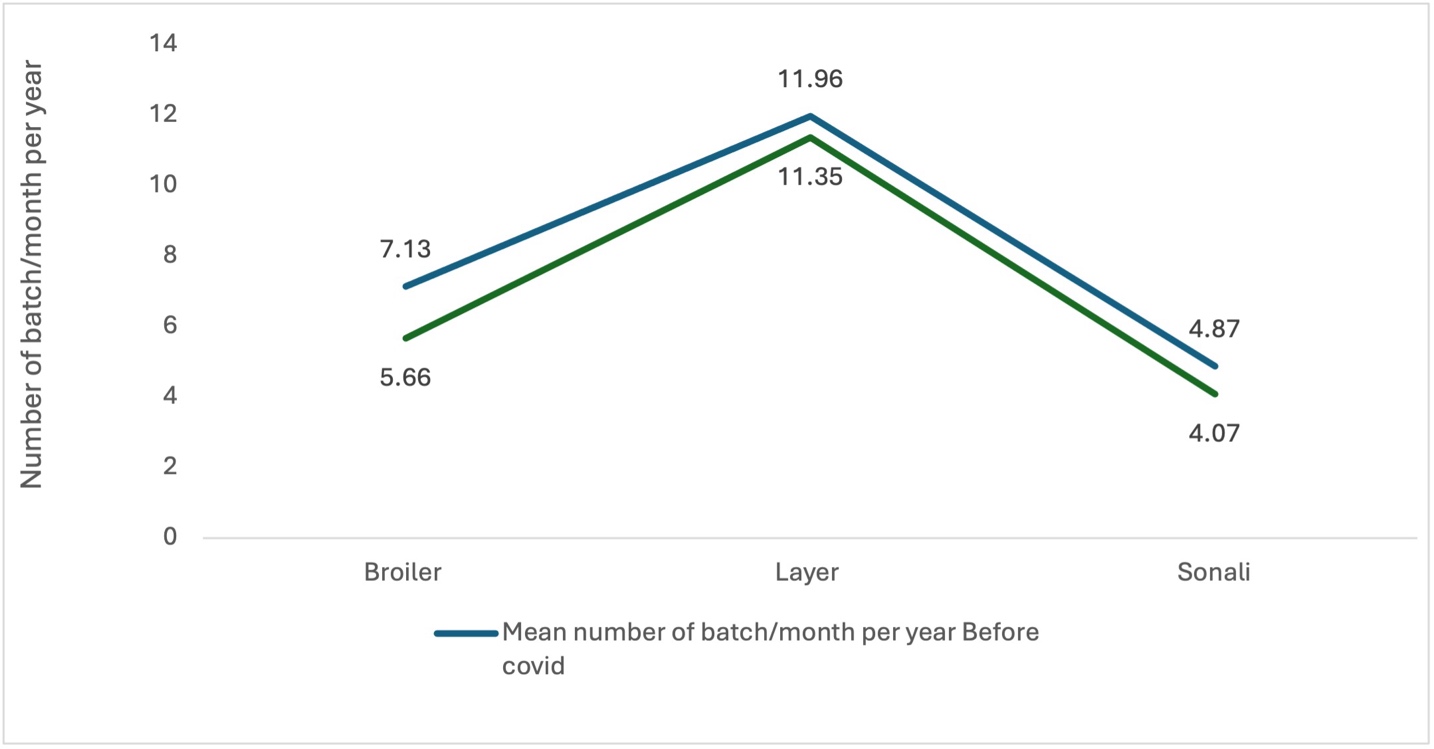
*

**Figure S1: Mean number of batches/months of farming per year before, during, and after the COVID-19 pandemic.**
